# Comparison of Clinical Characteristics and Outcome Between Vaccinated and Non-Vaccinated Patients of Covid-19 During the Delta Variant-Dominated Fourth Wave in a Tertiary Care Hospital in Karachi, Pakistan

**DOI:** 10.7759/cureus.23726

**Published:** 2022-04-01

**Authors:** Jamal Azfar Khan, Luqman Satti, Mahwash Bizanjo, Nadia A Ather

**Affiliations:** 1 Internal Medicine, Pakistan Navy Ship Shifa Hospital, Bahria University Medical & Dental College, Karachi, PAK; 2 Medical Microbiology, Pakistan Navy Ship Shifa Hospital, Bahria University Medical & Dental College, Karachi, PAK; 3 Internal Medicine/Infectious Diseases, Pakistan Navy Ship Shifa Hospital, Bahria University Medical & Dental College, Karachi, PAK; 4 Medicine, Sir Syed College of Medical Sciences, Karachi, PAK

**Keywords:** vaccination, treatment outcome, sars-cov-2, pakistan, delta variant, covid-19 vaccines, comorbidity

## Abstract

Introduction

In Pakistan, the fourth wave of coronavirus disease 2019 (Covid-19) started around July 2021, which was dominated by the Delta variant of the severe acute respiratory syndrome coronavirus 2 (SARS-CoV-2) virus. The vaccination drive to immunize the people of Pakistan against Covid-19 was also going on during this period. There were multiple types of vaccines being administered to the people of Pakistan, as the vaccines had been procured from multiple sources. Some people had apprehensions about different vaccines being administered in the country. The purpose of this study was to compare the clinical characteristics and outcome of the patients vaccinated against Covid-19 with those of the non-vaccinated patients during the fourth wave of Covid-19 in Pakistan Naval Ship (PNS) Shifa Hospital.

Methods

The cross-sectional descriptive study was performed at PNS Shifa Hospital Karachi, from July to October 2021. All the Covid-19 patients treated in PNS Shifa Hospital during the “fourth Covid-19 wave” were interviewed. Their medical records were accessed, and they were followed up till their discharge from the hospital. The vaccinated and non-vaccinated patients were compared for differences in their age or gender distribution, the severity of illness, comorbidities, and mortality.

Results

There were 884 participants in the study: 664 (75.11%) men and 220 (24.89%) women. There were 493 patients below 40 years of age, 233 were 40-59 years old, and 158 were aged 60 and above. One hundred and sixty-nine patients had one or more comorbidities, including hypertension, diabetes mellitus, ischemic heart disease, various malignancies, bronchial asthma, and chronic kidney disease. There were 63 (7.13%) obese patients, 28 of whom developed severe disease.

Five hundred and four (57%) patients were vaccinated and 380 (47%) were non-vaccinated. Among the vaccinated patients, the effect of Covid-19 was mild in 58.37%, moderate in 36.11%, severe in 0.79%, and critical in 4.37%. Among the non-vaccinated patients, the effect of Covid-19 was mild in 40.26%, moderate in 46.58%, severe in 3.16%, and critical in 10%. The difference in disease severity between the two groups was statistically significant (*p*<0.05).

Conclusion

Vaccinated Covid-19 patients had significantly lower severity of disease and displayed better outcomes when compared to non-vaccinated patients during the fourth Covid-19 wave dominated by the Delta variant of the SARS-CoV-2 virus.

## Introduction

Coronavirus disease 2019 (Covid-19) is an infectious disease spread by a novel severe acute respiratory syndrome coronavirus 2 (SARS-CoV-2) [1]. This pandemic has affected millions of people around the world in the last two years. Vaccines against Covid-19 have been developed by over 100 institutions around the world [2] because this pandemic would end either by developing herd immunity or by vaccinating a large majority of the world population.

Pakistan, like other countries in the world, responded to the Covid-19 pandemic by adopting various measures to curb the spread of this disease. It was a challenge to produce safe and effective vaccines in a short time to provide immunity to the maximum number of people. Pakistan was one of those countries which did not have the expertise or infrastructure to manufacture the Covid-19 vaccine to inoculate its population. Therefore, the government procured the vaccines from different sources to immunize the population.

Different approaches to Covid-19 vaccine development have been adopted around the world [3]. The vaccines which were available in regional vaccination centers in Karachi were: mRNA vaccines (Pfizer and Moderna), viral vector vaccines (AstraZeneca, CanSinoBIO, Pakvac, and Sputnik V), and inactivated (whole virus) vaccines (Sinopharm and Sinovac). The vaccination drive had already begun when there was a surge in Covid-19 cases in July 2021, resulting in its fourth wave in Pakistan. This wave was caused mainly by the (B.1.617.2) Delta variant of the SARS-CoV-2 virus [4]. By October 2021, 18% of the Pakistanis had been fully vaccinated against Covid-19 [5]. The administration of the booster shot had not started in the country by then.

The protective effect of various vaccine types on Pakistani patients infected with the Delta variant of Covid-19 has not been documented yet. This study fills this knowledge gap, as we present the comparison between vaccinated and non-vaccinated patients regarding their characteristics, disease severity, and outcome.

## Materials and methods

Study design and population

This a cross-sectional study, conducted in Pakistan Naval Ship (PNS) Shifa Hospital from July to October 2021.

Inclusion criteria

All the Covid-19 patients who attended the “Covid Clinic” or were admitted through the Emergency Department in PNS Shifa Hospital during the “fourth Covid-19 wave” were included in the study.

Exclusion criteria

The patients whose clinical presentation suggested Covid-19 but their polymerase chain reaction (PCR) test for Covid-19 came out negative were excluded. The patients who had been partially vaccinated against Covid-19, or had been diagnosed with Covid-19 within two weeks of getting the second dose of the vaccine were also excluded.

Sampling

Consecutive sampling was carried out.

Ethical review

The ethics review committee approved the study vide letter no. ERC/2021/MEDICINE/71.

Data collection

The researchers briefed the patients about the study and took consent from the patients to collect their data. The patients’ medical records were accessed for this purpose. Patients were interviewed by the researchers for any other information.

The vaccinated and non-vaccinated patients were compared for differences in their age or gender distribution, the severity of illness, comorbidities, and mortality. The severity of Covid-19 was categorized into mild, moderate, severe, and critical according to the National Institute of Health (NIH) criteria [6].

## Results

Eight hundred and eighty-four patients were diagnosed as Covid-19 patients in PNS Shifa Hospital during the study period, of which 493 (55.77%) patients were below 40 years old, 233 (26.36%) were 40-59 years old, and 158 (17.87%) were aged 60 and above. There were 664 (75.11%) men and 220 (24.89%) women. One hundred and sixty-nine (19.12%) patients had one or more comorbidities. The frequently occurring comorbidities were hypertension, diabetes mellitus, ischemic heart disease, various malignancies, bronchial asthma, and chronic kidney disease.

There were 63 (7.13%) cases with a BMI above 30. Of them, 28 patients (44.44%) developed a severe or critical Covid-19, whereas 48 of the 821 non-obese patients (5.85%) developed severe or critical Covid-19.

Of the total 884 cases, the effect of Covid-19 was mild in 449 (50.79%), moderate in 356 (40.27%), severe in 16 (1.81%), and critical in 60 (6.79%). Five hundred and four (57%) patients were vaccinated and 380 (47%) were non-vaccinated. The comparison of demographic distribution and comorbidities between vaccinated versus non-vaccinated patients is given in Table 1.

**Table 1:** Comparison of demographic distribution and comorbidities between vaccinated and non-vaccinated patients of Covid-19

	Vaccinated *n* (%)	Non-Vaccinated *n* (%)
Gender Male *n*=648	409 (63.12)	239 (36.88)
Female *n*=236	95 (40.25)	141 (59.75)
Age Below 40	319	174
40-59	115	118
60 and above	73	85
Comorbidities None	423	292
Hypertension	46	49
Diabetes mellitus	41	39
Ischemic heart disease	4	9
Bronchial asthma	4	3
Malignancy	6	2
Cerebrovascular accident	1	6
Chronic kidney disease	2	7

Disease severity and vaccine type

**Figure 1:**
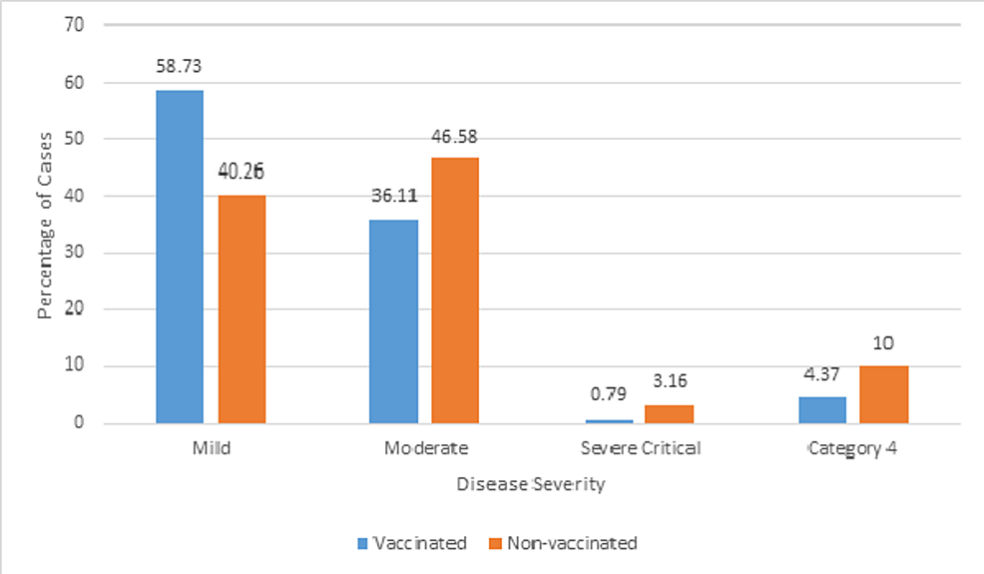
Comparison of disease severity between vaccinated and non-vaccinated patients of Covid-19

The breakdown of the severity of cases in patients who had been vaccinated against Covid-19 vis-à-vis the type of vaccine used is shown in Figure 1.

A chi-square test was carried out to examine the relationship between the vaccination status and the disease severity in Covid-19 patients. The relationship between these variables was significant (*p*<0.00001). The non-vaccinated patients suffered significantly more when compared to vaccinated patients.

## Discussion

Our study has shown that being vaccinated carries a lower probability of mortality and morbidity in Covid-19 patients, requiring few hospital admissions and mechanical ventilation, when compared to non-vaccinated patients. Five hundred and four cases were vaccinated by either inactivated, mRNA, or viral vector vaccines. Vaccinated cases (57% of total Covid-19 cases) were slightly more than the non-vaccinated cases (43%). This may be so because many people in Karachi had already been vaccinated against Covid-19. Although, more in number, more than half of them had a mild disease, only 5.16% vaccinated cases developed severe and critical disease when compared to 13.16% non-vaccinated patients. This shows that all the vaccines are potent enough to reduce the severity of the disease. These observations are consistent with other studies [7-9].

Several studies discussed the effectiveness of various covid-19 vaccines against hospitalization and the severity of the disease across the world [10-12]. However, there is a lack of local data and need to be documented. This study highlights the efficacy of Covid-19 vaccines, mainly inactivated, in reducing the number of hospitalization and minimizing the severity of the disease.

Most patients with Covid-19 were below 40 years. This is in contrast with the study which analyzed the age predominance in Europe [13]. According to this study, the authors observed higher prevalence in the 60 and above age group. Only 17.87% of patients were aged 60 and above, which could be because the Pakistani population is one of the youngest populations in the world [14]. The number of young patients may be higher when compared to the countries with older populations. Second, most of the people in the older age group have limited outdoor exposure. Old age, however, is associated with the severity of the disease [15,16]. In our cohort, we observed the same, as 31.6% of patients aged 60 and above developed severe and critical disease. The older patients have more comorbidities, which may be a compounding factor to get severe Covid-19.

We observed male preponderance in our study. Male predominance is seen globally as identified by various studies [13,17] as well as in Pakistan [18].

Many Covid-19 patients in our study had one or more comorbidities. It has been documented in various studies that the severity and mortality of Covid-19 are associated with chronic medical conditions [19]. Yang et al. conducted a meta-analysis to ascertain the comorbidities associated with Covid-19. They found that the most prevalent comorbidity of severe Covid-19 was hypertension [20]. Diabetes mellitus and cardiovascular diseases have also been documented as among the most common and consistent comorbidities in Covid-19 [21,22]. Our study corroborates these findings in Pakistani patients.

Chronic lung diseases such as bronchial asthma are supposed to be a risk factor of severe Covid-19 [23]. However, the relationship between Covid-19 and bronchial asthma is not well established clinically [24]. In our cohort, bronchial asthma was present only in eight patients (less than 1%), and this result was also in accordance with the published literature.

Obesity has been identified as a risk factor for severe disease, ICU admission, and high mortality in Covid-19 [25,26]. We also noted that a significant number of obese patients (44.44%) developed severe or critical disease.

Our study is unique in one aspect. It is probably the only study that compares the patient characteristics, the severity of disease, and clinical outcome of patients vaccinated with mRNA vaccines, inactivated virus vaccines, and viral vector vaccines. We could not find any other study with such a comparison in a single population in the medical literature.

We noted that very few participants in our study had been vaccinated by mRNA vaccines and that there were no patients with severe or critical illness in that group. This may lead the reader to conclude that mRNA vaccines result in fewer infections, thus providing more safety against Covid-19. This conclusion may be misguiding because the mRNA vaccines arrived much later in Pakistan, when compared to other vaccines, and in comparatively lesser quantity. So, the number of patients vaccinated with mRNA vaccines were fewer. Most of the population had been vaccinated with inactivated virus vaccine which had been imported from China and was the first to arrive in bulk in Pakistan.

We acknowledge the limitation in our study that it was a single-center research study. Hence, the results cannot be generalized to the population. We need multi-center research with bigger and diversified samples to document the effect of various vaccine types on the people of Pakistan. The emergence of new variants of the SARS-CoV-2 virus may limit the relevance of this study in the future.

## Conclusions

The Pakistani population is being vaccinated against Covid-19 with the help of multiple types of vaccines. Some people prefer one type of vaccine over the others, usually based upon the country of origin of the vaccine and some people are also hesitant to get vaccinated. These attitudes are not based on any evidence. This study shows that vaccination against Covid-19 has been effective in reducing the severity of Covid-19 as well as the mortality associated with it, irrespective of the type of vaccine used. This study may help in addressing vaccine hesitancy and improve the coverage of the ongoing vaccination campaign in Pakistan and elsewhere.

## References

[1] Haslak F, Gunalp A, Cebi MN (2022). Early experience of COVID-19 vaccine-related adverse events among adolescents and young adults with rheumatic diseases: a single-center study. Int J Rheum Dis.

[2] Wang J, Peng Y, Xu H, Cui Z, Williams RO III (2020). The COVID-19 vaccine race: challenges and opportunities in vaccine formulation. AAPS PharmSciTech.

[3] Chowdhury MR, Islam S, Matin MN (2021). COVID-19 vaccine race: an overview and update. JDDT.

[4] (2022). Pakistan Humanitarian Situation Report No. 28. https://www.unicef.org/media/107031/file/%20Pakistan-Humanitarian-sitRep-No28-31-August-2021.pdf.

[5] (2022). Coronavirus pandemic (COVID-19). https://ourworldindata.org/coronavirus.

[6] (2022). Clinical spectrum of SARS-CoV-2 infection. (2021). https://www.covid19treatmentguidelines.nih.gov/overview/clinical-spectrum/.

[7] Jara A, Undurraga EA, González C (2021). Effectiveness of an inactivated SARS-CoV-2 vaccine in Chile. N Engl J Med.

[8] Tenforde MW, Self WH, Adams K (2021). Association between mRNA vaccination and COVID-19 hospitalization and disease severity. JAMA.

[9] Muthukrishnan J, Vardhan V, Mangalesh S (2021). Vaccination status and COVID-19 related mortality: a hospital based cross sectional study. Med J Armed Forces India.

[10] Sadoff J, Gray G, Vandebosch A (2021). Safety and efficacy of single-dose Ad26.COV2.S vaccine against Covid-19. N Engl J Med.

[11] Baden LR, El Sahly HM, Essink B (2021). Efficacy and safety of the mRNA-1273 SARS-CoV-2 vaccine. N Engl J Med.

[12] Fadlyana E, Rusmil K, Tarigan R (2021). A phase III, observer-blind, randomized, placebo-controlled study of the efficacy, safety, and immunogenicity of SARS-CoV-2 inactivated vaccine in healthy adults aged 18-59 years: an interim analysis in Indonesia. Vaccine.

[13] Cannistraci CV, Valsecchi MG, Capua I (2021). Age-sex population adjusted analysis of disease severity in epidemics as a tool to devise public health policies for COVID-19. Sci Rep.

[14] Burki SJ (2005). Educating the Pakistani masses. Education reform in Pakistan.

[15] Gallo Marin B, Aghagoli G, Lavine K (2021). Predictors of COVID-19 severity: a literature review. Rev Med Virol.

[16] Wang Y, Zhou Y, Yang Z, Xia D, Hu Y, Geng S (2020). Clinical characteristics of patients with severe pneumonia caused by the SARS-CoV-2 in Wuhan, China. Respiration.

[17] Peckham H, de Gruijter NM, Raine C (2020). Male sex identified by global COVID-19 meta-analysis as a risk factor for death and ITU admission. Nat Commun.

[18] Zia S (2020). COVID-19 and gender infectivity-mortality rate among Pakistani population. Working with Older People.

[19] Rod JE, Oviedo-Trespalacios O, Cortes-Ramirez J (2020). A brief-review of the risk factors for covid-19 severity. Rev Saude Publica.

[20] Yang J, Zheng Y, Gou X (2020). Prevalence of comorbidities and its effects in patients infected with SARS-CoV-2: a systematic review and meta-analysis. Int J Infect Dis.

[21] Papadokostaki E, Tentolouris N, Liberopoulos E (2020). COVID-19 and diabetes: what does the clinician need to know?. Prim Care Diabetes.

[22] Mozzini C, Cicco S, Setti A (2021). Spotlight on cardiovascular scoring systems in Covid-19: severity correlations in real-world setting. Curr Probl Cardiol.

[23] Chhiba KD, Patel GB, Vu TH (2020). Prevalence and characterization of asthma in hospitalized and nonhospitalized patients with COVID-19. J Allergy Clin Immunol.

[24] Liu S, Zhi Y, Ying S (2020). COVID-19 and asthma: reflection during the pandemic. Clin Rev Allergy Immunol.

[25] Soeroto AY, Soetedjo NN, Purwiga A (2020). Effect of increased BMI and obesity on the outcome of COVID-19 adult patients: a systematic review and meta-analysis. Diabetes Metab Syndr.

[26] Huang Y, Lu Y, Huang YM (2020). Obesity in patients with COVID-19: a systematic review and meta-analysis. Metabolism.

